# Modeling the origins of mammalian sociality: moderate evidence for matrilineal signatures in mouse lemur vocalizations

**DOI:** 10.1186/1742-9994-11-14

**Published:** 2014-02-20

**Authors:** Sharon E Kessler, Ute Radespiel, Alida I F Hasiniaina, Lisette M C Leliveld, Leanne T Nash, Elke Zimmermann

**Affiliations:** 1 Arizona State University, School of Human Evolution and Social Change, Box 872402, Tempe, AZ 85287-2402, USA; 2 University of Veterinary Medicine Hannover, Institute of Zoology, Bünteweg 17, 30559 Hannover, Germany; 3 Faculté des Sciences Université de Mahajanga, BP 652 Mahajanga, Madagascar; 4 Leibniz Institute for Farm Animal Biology (FBN), Institute for Behavioural Physiology, Wilhelm-Stahl-Allee 2, D-18196 Dummerstorf Germany

**Keywords:** Acoustic signature, Maternal kin, Solitary forager, Ancestral primate, Microsatellite

## Abstract

**Introduction:**

Maternal kin selection is a driving force in the evolution of mammalian social complexity and it requires that kin are distinctive from nonkin. The transition from the ancestral state of asociality to the derived state of complex social groups is thought to have occurred via solitary foraging, in which individuals forage alone, but, unlike the asocial ancestors, maintain dispersed social networks via scent-marks and vocalizations. We hypothesize that matrilineal signatures in vocalizations were an important part of these networks. We used the solitary foraging gray mouse lemur (*Microcebus murinus*) as a model for ancestral solitary foragers and tested for matrilineal signatures in their calls, thus investigating whether such signatures are already present in solitary foragers and could have facilitated the kin selection thought to have driven the evolution of increased social complexity in mammals. Because agonism can be very costly, selection for matrilineal signatures in agonistic calls should help reduce agonism between unfamiliar matrilineal kin. We conducted this study on a well-studied population of wild mouse lemurs at Ankarafantsika National Park, Madagascar. We determined pairwise relatedness using seven microsatellite loci, matrilineal relatedness by sequencing the mitrochondrial D-loop, and sleeping group associations using radio-telemetry. We recorded agonistic calls during controlled social encounters and conducted a multi-parametric acoustic analysis to determine the spectral and temporal structure of the agonistic calls. We measured 10 calls for each of 16 females from six different matrilineal kin groups.

**Results:**

Calls were assigned to their matriline at a rate significantly higher than chance (pDFA: correct = 47.1%, chance = 26.7%, p = 0.03). There was a statistical trend for a negative correlation between acoustic distance and relatedness (Mantel Test: g = -1.61, Z = 4.61, r = -0.13, p = 0.058).

**Conclusions:**

Mouse lemur agonistic calls are moderately distinctive by matriline. Because sleeping groups consisted of close maternal kin, both genetics and social learning may have generated these acoustic signatures. As mouse lemurs are models for solitary foragers, we recommend further studies testing whether the lemurs use these calls to recognize kin. This would enable further modeling of how kin recognition in ancestral species could have shaped the evolution of complex sociality.

## Introduction

Maternal kin selection (the preferential treatment of matrilineal relatives [1,2]) has been argued to be one of the driving forces in the evolution of mammalian sociality, underpinning some of the most complex and intriguing social behaviors including communal infant rearing and socialization, the evolution of group-living, alliance formation and cooperation [1,3-5]. While such manifestations of kin selection are well documented in gregarious species that live in complex social groups [3,4], its evolutionary foundations are likely to have emerged in less complex, ancestral species ([6], but see [7]). Given that ancestral mammals are believed to have been asocial with no social relationships maintained outside of mating and rearing infants [6], tracing how maternal kin selection may have formed the backbone for this transition is likely to be crucial to understanding how social complexity evolves.

A prerequisite of maternal kin selection in any mammalian social system is that maternal kin must be sufficiently distinctive from nonkin that they can be recognized and thus receive preferential treatment [1,2]. For the asocial and nocturnal ancestral mammals [6], this would have also meant being distinctive over distances, through darkness, and dense foliage where visual and olfactory cues would have been inefficient. Mammals under these conditions would be expected to benefit from having matrilineal signatures in their vocalizations.

To date, much of the attention that has been given to investigating matrilineal signatures in mammalian vocalizations has focused on social species (ie. goats [8,9], meerkats [10], marmots [11], sperm whales and killer whales [12-14], bats [15-17] and the socially variable house mouse [18,19]). Much less has been done on solitary species (i.e., pandas [20]). In the solitary pandas, individual signatures were found, but there was no correlation between overall acoustic distance between individuals and their relatedness, and only a few individual parameters correlated with relatedness [20]. Though the authors did not clarify whether relatedness was matrilineal, patrilineal, or both, the lack of stronger results may still indicate that pressure to encode kinship within vocalizations may not be as strong as in the more social species [20]. Each of these studies that investigated kin signatures, either exclusively focused on matrilineal relatedness or had a high likelihood of relatedness from both patrilineal and matrilineal relationships, thus suggesting that matrilineal relatedness had a strong role in the signatures found. We differentiate between *individual* signatures that may be recognized by kin (i.e., primates: [21-24], pinnipeds [25], elephants [26], dolphins [27]) and *matrilineal* signatures. Matrilineal signatures have the important distinction that they may enable the recognition of unfamiliar maternal kin via the similarity to known maternal kin, thus facilitating the preferential treatment of unfamiliar maternal kin.

In order to better understand the evolutionary transition from asociality to social complexity, we focus on primates, an order in which some lineages have evolved highly complex, cohesive social groups while other lineages are believed to have retained the social system that is believed to be ancestral to primates: solitary foraging [6]. In the lineages that evolved social systems with cohesive social groups, the ancestral solitary foragers are believed to have been a transition phase between asociality and group-living [6]. Solitary foragers forage alone, but maintain a dispersed social network of relationships with conspecifics communicating through vocalizations and scent-marks, and often have consistent co-sleeping associations [6]. It is these dispersed social networks in ancestral primates that are thought to have been the foundation for the evolution of more complex primate social systems [6], thus they are likely to have been crucial for kin networks and a likely pathway for kin selection [28,29].

In order to determine whether matrilineal signatures in vocalizations may have facilitated matrilineal kin selection in solitarily foraging ancestral primates, we use the gray mouse lemur (*Microcebus murinus*) to model ancestral primates. Mouse lemurs are frequently used as ancestral primate models because their socioecology is thought to be similar to that of the last common ancestor of the primate order [6,30-46]. Like mouse lemurs today, ancestral primates are thought to have been small-bodied, small-brained nocturnal solitary foragers that forage for fruits and insects in the thin, terminal ends of branches [6,30-46]. Therefore, we use the dispersed social networks of living mouse lemurs to model ancestral primate social organization and to reconstruct the social behavior patterns from which present-day primate diversity evolved [6].

Our current knowledge of the gray mouse lemur’s dispersed social networks makes it an excellent model species in which to test for matrilineal signatures. Male dispersal and female philopatry are common [47,48]. Both sexes forage solitarily in home ranges that overlap with those of other individuals of both sexes [43,49]. During the day, adult males sleep alone [44,50]. Females form sleeping groups with female kin and cooperatively raise their young in tree holes [42,50]. Immature males and females are socialized within these groups [42,50] and thus have ample opportunity to hear and learn the calls of their matrilineal kin. However, given that larger nest groups may split, it is also possible for subsequent generations to encounter matrilineal kin with whom they personally did not share a nest [42]. It is also possible that inherited vocal tract morphology (see source-filter theory: i.e., [51-53]) could cause related individuals to produce similar calls. Thus, both genetic factors and social learning could contribute to the development of matrilineal signatures in this species.

**Figure 1:**
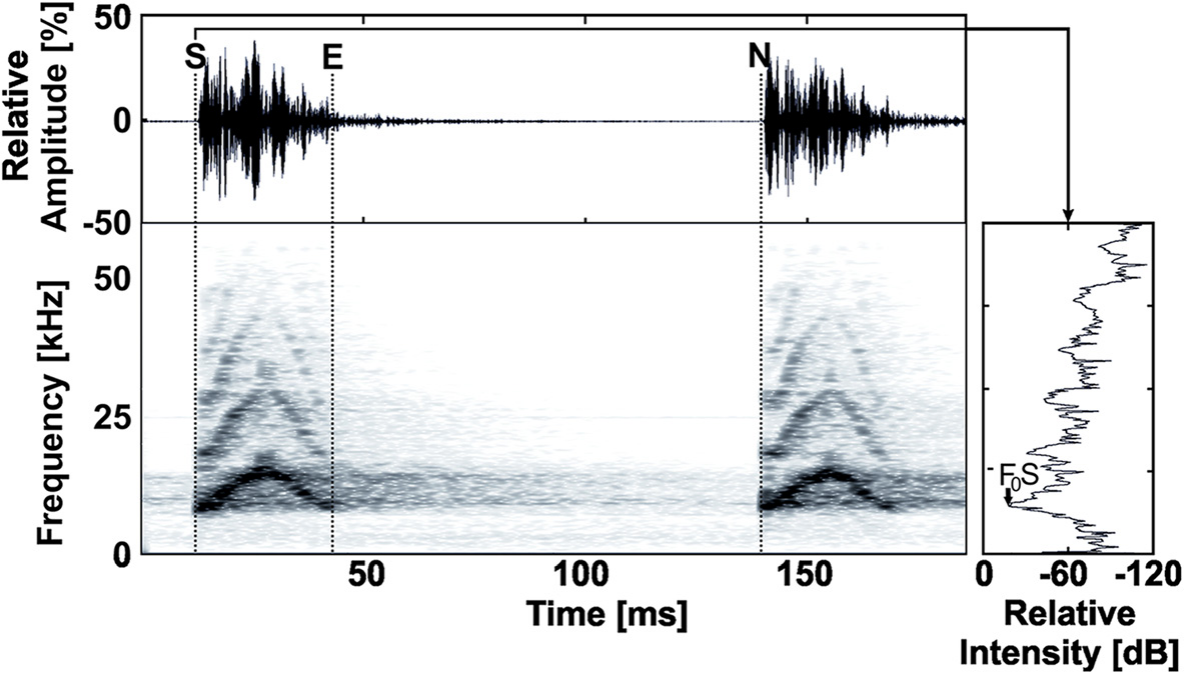
**An oscillogram, spectrogram and power spectrum depicting some of the acoustic parameters of the agonistic call.** S, E, and N show the start time, end time, and start of the next call, respectively. F_0_S shows the measurement of the fundamental frequency on the power spectrum. Figure produced in BatSound Pro 3.31 (Pettersson Elektronik AB, Upsala Sweden) according to [58]. For more information see Table 1 or Leliveld et al. [58].

Mouse lemurs have an elaborate vocal repertoire and use vocalizations in a diversity of social interactions (e.g., mating contexts [54,55], mother-infant communications [56], emotional state [5,57], paternal kin recognition [28]). We chose to investigate the individually distinctive agonistic call [58]. It is a short, frequency modulated vocalization with an upward and downward sweep (Figure 1) containing harmonics in both the audible and ultrasonic range [58]. Because aggressive/defensive encounters have the potential to be very costly due to injuries sustained, we predicted that it would be advantageous for agonistic calls to contain matrilineal signatures so that aggression amongst matrilineal relatives could be minimized. We hypothesized that these agonistic calls will be distinctive by matrilineal kin group and that the genetic relatedness of female dyads will negatively correlate with their acoustic distance. We found moderate evidence for matrilineal signatures and a trend suggesting that increasing relatedness is associated with decreasing acoustic distance. Further studies are needed to determine whether mouse lemurs use these signatures to recognize kin.

## Results

### Acoustic differences in agonistic calls between matrilines

Qualitative differences are visible between matrilines (Additional file 1) in both frequency and temporal parameters. For example, several individuals in matriline 6 gave calls with an unusually high peak frequency, matriline 5 gave calls of longer duration, and matrilines 1 and 3 typically gave lower frequency calls, with the calls of matriline 1 being generally qualitatively shorter than those of 3.

The principal component analysis produced two components which together explained 66.2% of the variation in the original dataset. The first component was highly correlated (>0.4 or < -0.4) with all original acoustic parameters, but correlated most strongly (>0.7) with the frequency parameters, and thus, it is referred to as the frequency component (48.6% of the total variation). The second component correlated highly (>0.4 or < -0.4) with call duration and inter-call interval and is thus called the time component (17.5% of the total variation). Table 1 shows the 25% quartile, median, and 75% quartile for each of the original acoustic parameters and their loadings on the frequency component and the time component. Table 2 shows the matrix of pairwise acoustic distances calculated for each dyad of females.

**Table 1:** The 25%, 50%, and 75% quartiles of the original acoustic parameters and the loadings for each parameter on the frequency (component 1) and time (component 2) components

**Parameters**		**Quartiles**		**Component loadings**	
	**25%**	**Median**	**75%**	**Component 1**	**Component 2**
F_0_S (Hz)	10156	11133	12061	**0.828**	0.330
F_0_Peak (Hz)	12500	13770	16602	**0.785**	0.064
F_0_E (Hz)	10156	11523	12891	**0.805**	-0.253
Start Bandwidth (Hz)	3062	3749	4646	**0.590**	0.263
Call Duration (ms)	32	40	48	**-0.624**	**0.658**
Time to Peak (ms)	17	20	26	**-0.696**	0.280
Inter-call Interval (ms)	101	148	197	**-0.481**	**-0.685**

Parameters classified as highly loaded (>0.4 or < -0.4) are shown in bold.

**Table 2:** **The top matrix shows the pairwise relatedness values between females according to Queller and Goodnight**[59,60]

	**06-09**	**10-10**	**11-11**	**101-10**	**112-10**	**113-10**	**17-10**	**19-10**	**28-09**	**36-11**	**41-11**	**45-10**	**46-11**	**51-10**	**52-11**	**58-10**
**06-09**		0.11	0.25	-0.18	0.03	-0.15	-0.17	**0.61**^**a**^	-0.22	-0.20	**0.29**^**b**^	0.12	0.05	0.12	-0.14	0.09
**10-10**	0.38		0.04	-0.08	**0.41**^**a**^	-0.02	-0.15	0.10	-0.03	-0.09	0.00	-0.02	0.00	-0.02	-0.02	0.01
**11-11**	1.27	1.65		0.06	0.28	0.05	-0.17	0.24	-0.22	-0.03	-0.03	0.14	0.16	**0.34**^**c**^	-0.04	**0.62**^**a**^
**101-10**	0.14	0.24	1.41		0.08	0.01	-0.10	-0.20	0.00	-0.05	0.03	**0.51**^**a**^	-0.22	0.01	0.16	-0.12
**112-10**	1.00	**0.62**	2.27	0.85		-0.01	-0.12	-0.06	0.06	-0.07	-0.14	0.08	0.09	-0.09	0.07	0.09
**113-10**	0.15	0.23	1.42	0.01	0.85		-0.05	-0.08	-0.02	0.01	0.19	0.09	0.11	0.09	**0.36**^**a**^	0.02
**17-10**	1.96	2.34	0.69	2.10	2.95	2.11		-0.28	**0.52**^**a**^	**0.49**^**a**^	-0.02	-0.14	**0.44**^**a**^	-0.05	-0.04	-0.19
**19-10**	**0.08**	0.46	1.19	0.22	1.08	0.23	1.88		-0.31	-0.05	**0.44**^**a**^	0.10	-0.06	0.20	-0.24	0.16
**28-09**	1.02	1.40	0.25	1.16	2.01	1.17	**0.94**	0.94		**0.23**^**b**^	-0.08	-0.19	**0.17**^**b**^	-0.02	0.06	-0.15
**36-11**	2.82	3.20	1.55	2.96	3.82	2.97	**0.87**	2.74	**1.81**		-0.06	-0.08	**0.38**^**a**^	0.10	0.01	-0.05
**41-11**	**0.50**	0.89	0.77	0.65	1.50	0.65	1.45	**0.42**	0.51	2.32		0.28^c^	0.03	0.10	-0.08	-0.01
**45-10**	0.17	0.55	1.10	**0.31**	1.17	0.32	1.79	0.09	0.85	2.65	0.33		0.01	0.09	-0.10	-0.03
**46-11**	0.07	0.45	1.20	0.21	1.07	0.22	**1.89**	0.01	**0.95**	**2.75**	0.43	0.10		-0.09	0.01	0.08
**51-10**	0.95	1.33	**0.32**	1.09	1.94	1.10	1.01	0.87	0.07	1.88	0.44	0.78	0.88		-0.19	**0.29**^**b**^
**52-11**	0.38	0.77	0.88	0.53	1.38	**0.53**	1.57	0.31	0.63	2.44	0.12	0.22	0.32	0.56		-0.07
**58-10**	0.42	0.81	**0.85**	0.57	1.42	0.57	1.53	0.34	0.59	2.40	0.08	0.25	0.35	**0.52**	0.04	

The bottom matrix shows the acoustic distances for the female dyads. In both matrices, bold values show dyads from the six kin groups (compare Table 3).

^a^P < 0.001, Likelihood ratio > 37.02, Type II error <0.77.

^b^P < 0.01, Likelihood ratio > 11.95, Type II error <0.59.

^c^P < 0.05, Likelihood ratio > 3.31, Type II error <0.36.

### Genetic relatedness

**Figure 2:**
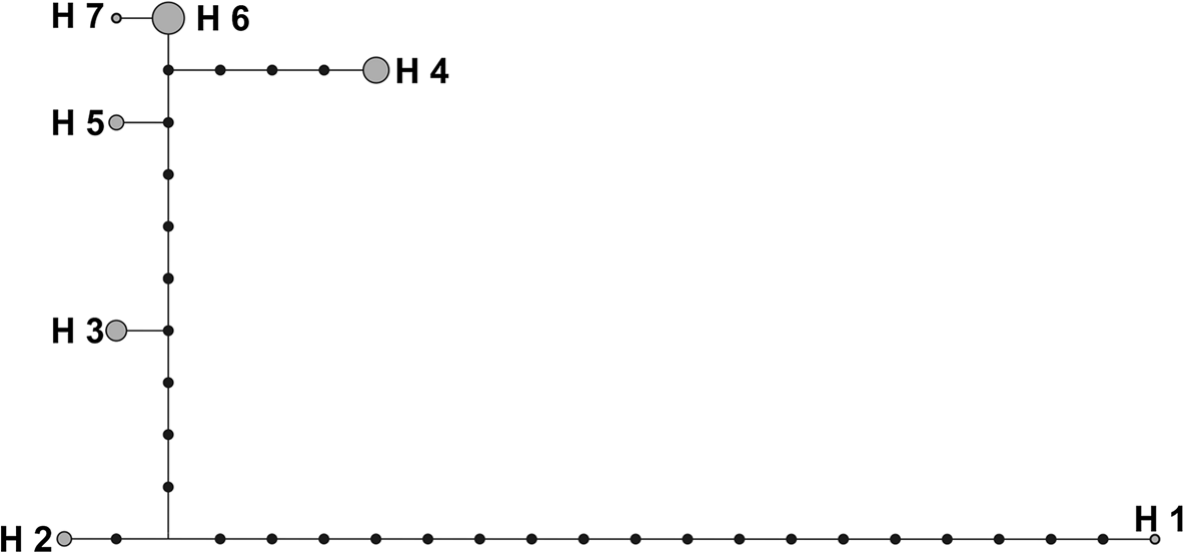
**A mitochondrial D-loop haplotype network of the population.** Kin groups 1-3 are from haplotype 6. Groups 4 and 5 are from haplotype 3 and group 6 is from haplotype 4.

Median pairwise relatedness for all dyads in the population is r = -0.02 (n = 107 individuals, min = -0.38, max = 0.91). Median pairwise relatedness for the females within the kin groups was r = 0.41 (n = 16 females, min = 0.30, max = 0.52, Table 3), whereas the between kin group median relatedness was r = -0.02 (n = 16 females, min = -0.12, max = 0.06). Table 2 shows the matrix of pairwise relatedness values of all the females in the kin groups. Within the females in the population, we found seven mitochondrial haplotypes (Figure 2). The kin groups in this study belonged to the three most frequent haplotypes (H3, H4, H6).

**Table 3:** The six kin groups, their co-sleeping behavior, relatedness values calculated from seven microsatellites, allelic exclusions from the microsatellites (number of loci with no shared alleles), and the mitochondrial d-loop haplotype

**Kin group**	**Dyad**	**Co-sleep**	**Relatedness**	**Allelic exclusions**	**Haplotype**
1 (n = 3)	06-09 & 19-10	No	0.61^a^	0	06-09: H6
	06-09 & 41-11	??	0.29^b^	1	41-11: H6
	19-10 & 41-11	Yes	0.44^a^	0	19-10: ??
2 (n = 3)	51-10 & 58-10	Yes	0.29^b^	0	All: H6
	51-10 & 11-11	Yes	0.34^c^	2	
	58-10 & 11-11	Yes	0.62^a^	0	
3 (n = 2)	10-10 & 112-10	Yes	0.41^a^	0	All: H6
4 (n = 2)	45-10 & 101-10	Yes	0.51^a^	0	All: H3
5 (n = 2)	113-10 & 52-11	No	0.36^a^	0	All: H3
6 (n = 4)	28-09 & 17-10	Yes	0.52^a^	0	All: H4
	28-09 & 36-11	??	0.23^b^	3	
	28-09 & 46-11	??	0.17^b^	2	
	17-10 & 36-11	Yes	0.49^a^	0	
	17-10 & 46-11	Yes	0.44^a^	0	
	36-11 & 46-11	Yes	0.38^a^	1	

?? means data not available. Allelic exclusions were included to faciliatate comparisons with previous genetic analyses on sleeping groups in this population of mouse lemurs (e.g., [42]).

^a^P < 0.001, Likelihood ratio > 37.02, Type II error <0.77.

^b^P < 0.01, Likelihood ratio > 11.95, Type II error <0.59.

^c^P < 0.05, Likelihood ratio > 3.31, Type II error <0.36.

### Kin group signatures and correlation between acoustic distance and genetic relatedness

**Figure 3:**
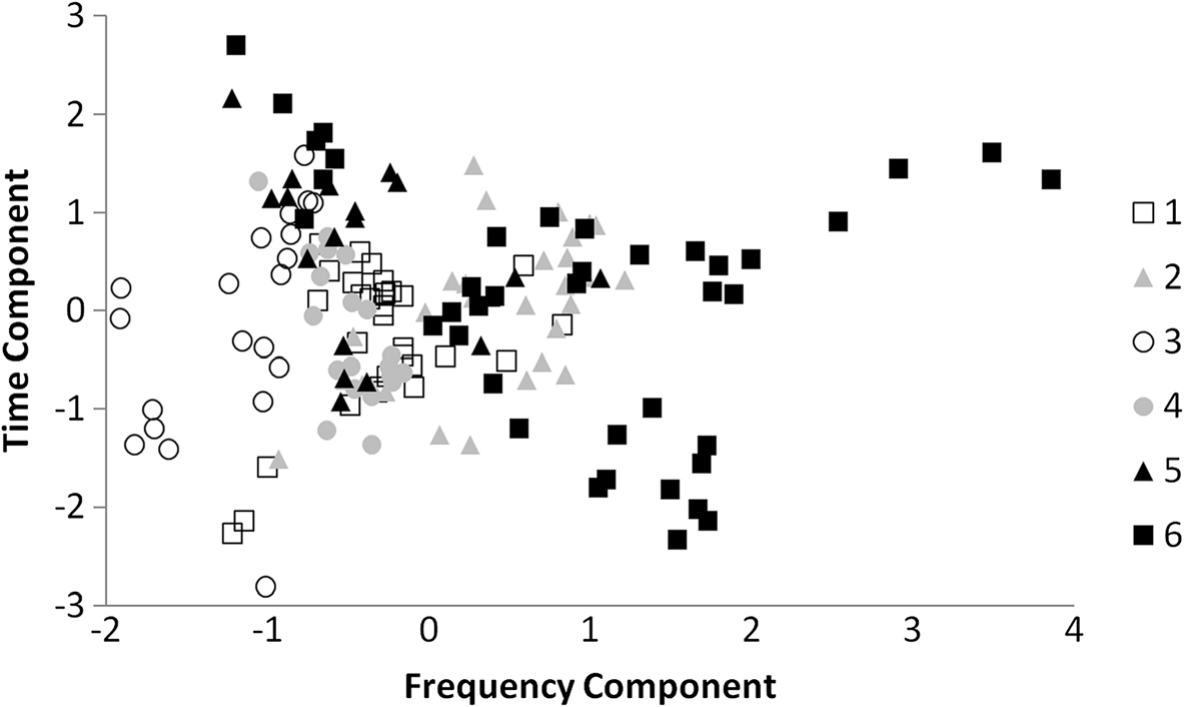
**A scatterplot showing the separation of the kin groups produced by the frequency and time components of the principal component analysis.** Individual symbols each represent one of the 160 analyzed calls.

**Figure 4:**
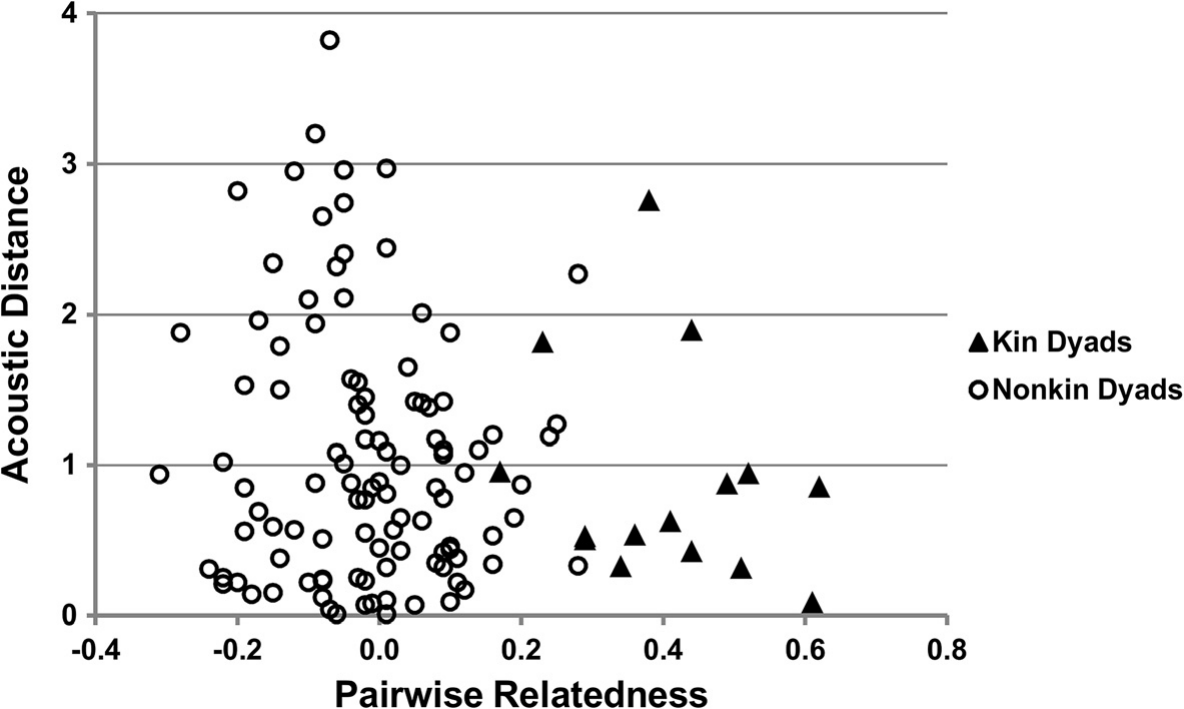
A scatterplot showing a weak negative relationship between pairwise genetic relatedness (X axis) and acoustic distance between dyads (Y axis).

The pDFA correctly classified 47.1% of the 160 calls by kin group (pDFA, chance level = 26.7%, p = 0.03). Figure 3 shows the separation of the kin groups produced by the frequency and time components (classification table produced by a non-permutated DFA is presented in Additional file 2). In addition, we found a statistical trend for a weak, negative correlation between genetic relatedness and acoustic distance among the 16 females (Mantel Test, g = -1.61, Z = 4.61, r = -0.13, p = 0.058, Figure 4). Thus, an increase in relatedness was associated with a tendency towards a decrease in acoustic distance.

## Discussion

We found moderate evidence for matrilineal signatures in mouse lemur agonistic calls. While the calls were classified to the correct matriline at a rate significantly higher than chance (47% correct vs. 26.7% chance), the false classification was still made more than half the time. In addition, while we found a statistical trend for a negative relationship between genetic relatedness and acoustic distance, the correlation coefficient was relatively low.

Given that the dispersed matrilineal social system of mouse lemurs provides the opportunity for matrilineal signatures to occur through both inherited traits in vocal morphology (see source filter theory, i.e., [51-53]) and through social learning [42,50], both may have been important proximate mechanisms for the moderate signatures found in this study. Offspring may inherit vocal tract morphology affecting vocal cord length and thickness which would in turn affect the fundamental frequency of the calls [51-53]. And indeed, fundamental frequency parameters were important in this analysis, loading highly on principle component 1. Offspring could also inherit traits having to do with lung capacity which could influence breathing rate and thus affect acoustic parameters such as call duration and inter-call interval (see source filter theory, i.e. [51-53]), both of which loaded highly on component 2. Unfortunately, it was not possible to collect measurements of vocal tract morphology (ie, length and thickness of vocal folds, length of vocal tract) as this would be highly invasive and, in the field, very complicated. Thus, it is not possible for us to test for a relationship between vocal tract morphology and acoustics. Furthermore, we do not expect less targeted morphological measures (ie. body mass, body length, head size, etc) to be useful proxies for heritability of vocal tract morphology, because they will often vary with pregnancy status, age, season, and the availability of sufficient nutrition for the developing females, none of which we could control for in this population of wild mouse lemurs. Additionally, previous reviews have shown that body size tends to correlate with acoustic differences across age and sex classes in monkeys and humans, but within those classes the relationship is less clear [52,61].

In addition to genetic mechanisms, it is possible that offspring may also learn to produce calls similar to the calls of the matrilineal relatives from the same nest, which they hear during socialization [42,50]. Prior research has shown that infant mouse lemurs produce highly variable infant calls that then stabilize into the adult form around the time of weaning [62]. This could mean that social learning during development may be crucial for the development of kin signatures. Similar findings have been found in birds [63] and other mammals (i.e., [8]). Unfortunately our data do not allow us to separate the effects of social learning and heritability. While co-sleeping promotes familiarity and thus generates opportunities for social learning, we cannot reliably compare co-sleepers with non-co-sleepers. We cannot exclude the possibility that the non-co-sleeping dyads may have co-slept when they were younger, but no longer did during our study. This is particularly likely for 28-09 and 36-11 and for 28-09 and 46-11 who were not observed to co-sleep. However, since 28-09 was not recaptured in the second year of the study, we do not know if she was still alive. If she was still alive when 36-11 and 46-11 were born, she may have co-slept with them until her death. Similarly, 06-09 was not captured the second year of the study when 41-11 was first caught, thus we do not know if both members of this dyad were alive at the same time. In addition, 19-10 and 06-09 were both at least one year old at the start of the study. Thus, they could be a sibling dyad or mother-daughter dyad which was part of a sleeping group which split as the lemurs aged. However, while it is not possible to distinguish between the two mechanisms here (genetics and social learning), we suspect that the two proximate mechanisms are not mutually exclusive and may even have additive effects (though additional interaction effects could also be possible). Thus, we expect that both mechanisms are likely to have contributed to the evolution of the moderate matrilineal signatures present in the calls.

While these matrilineal signatures are statistically present, their weakness brings up several intriguing questions. The first is whether the lemurs would be expected to use them to recognize kin. Prior work on mouse lemurs showed that females responded differently to calls from paternal kin and nonkin when the classification rate was 79% (mating calls), but not when it was 45% (alarm calls) [28]. Though our current study focuses on only the agonistic calls, future work testing other call types for matrilineal signatures would be very valuable. It would contribute to an increasing body of literature which suggests that the strength of acoustic signatures and the corresponding responses from conspecifics varies greatly by call type (i.e., [21,23,58,64,65]). However, while playback studies like those above focus exclusively on acoustic cues, in the wild kin recognition is a multi-modal process and the relative strength of each of the cue types may vary depending upon the context in which selection is expected to occur. It is possible that weaker signatures might be present in calls typically used at short distances when visual and olfactory cues would also be available [58,65]. Given that agonistic calls are frequently given during close-range conflicts, and mouse lemurs have not been documented to recruit kin for alliances, it is possible that kin signatures in agonistic calls may not be under strong selection (though see [66] for a case of nonkin recruitment).

As solitary foragers are thought to be the intermediary link between the solitary ancestral mammalian condition and the more complex, derived forms of gregarious primate sociality [6], our results suggest that ancestral solitary foragers may also have had moderate matrilineal signatures in their vocalizations. Such signatures, if used for kin recognition, may have been a crucial element of the dispersed social networks from which more complex, gregarious sociality is thought to have evolved in primates. However, if these signatures are not recognized, then it would lend support to an alternative theory of cryptic kin selection [67] in which kin-based sociality is thought to have evolved from the spatial proximity of kin alone. As mouse lemur females (as well as females of many other species [68]) are philopatric, they could interact preferentially with kin simply because kin are there, rather than because they discriminate kin and nonkin, and this could then be the foundation from which more complex forms of kin-based sociality evolved [67]. Future work is underway to test for the vocal recognition of matrilineal kin in this population and is expected to facilitate testing hypotheses about the possible influences of kin-biased behavior on the evolution of complex sociality (ie., [7]).

The presence of both matrilineal and individual signatures in several species (i.e., the gray mouse lemur [58], bats [15]) brings up the question of whether different pressures select for kin vs. individual signatures [2,69,70]. It could be that there is an optimal amount of divergence between individuals within a kin group which optimizes both types of signatures, thus enabling an individual to be categorized by kin group and be simultaneously individually distinctive within its kin group [12,71]. Or, it is possible that one of the two levels of distinctiveness is more strongly targeted by selection and that the other is merely a side effect of inherited vocal tract morphology and social learning of call production [2]. For example, if individual distinctiveness is highly selected for, how distinctive an individual could be might be constrained by inherited vocal tract morphology and socially learned call production [2]. Alternatively, if kin group distinctiveness is highly selected for, within kin group similarity might be constrained by their individual genetics and individual learning experiences. To tease the two apart, future work should compare the acoustic distances between individuals within kin groups across species with different social systems. Future work should also examine whether females use these moderate signatures to discriminate familiar kin, unfamiliar kin, and familiar nonkin. If only familiar kin are recognized, then it would suggest that the recognition of kin occurs primarily through familiarity with individuals who happen to be kin (see [63] for work on cooperatively breeding birds). Such future work, testing whether the lemurs actually recognize matrilineal kin will be highly important to determining the biological and evolutionary significance of these signatures.

## Conclusions

We found moderate evidence for matrilineal signatures in mouse lemur agonistic calls. In addition, there was a tendency for acoustic distance between individuals to decrease as relatedness increased. We expect that both inherited morphological traits and social learning are proximate mechanisms for these signatures. Given that mouse lemurs are solitary foragers, they serve as models for the ancestral solitary foragers that are believed to have been the link between ancestral solitary mammals and derived, more complex forms of sociality in primates [6]. Thus, our results suggest that the ancestral solitary foragers might have had similar, moderate, matrilineal signatures in their calls and we recommend further studies testing whether the lemurs use these calls recognize kin. Such studies would enable further modeling of how kin recognition in ancestral primates might have impacted the evolution of more complex forms of sociality in primates.

## Methods

### Field site and animal housing

This study was conducted at the Ankarafantsika National Park near the Ampijoroa forestry station (16^○^19’S, 46^○^48’E) in northwestern Madagascar during the dry seasons (May through November) of 2010 and 2011 in the designated research area of the park called Jardin Botanique A (JBA). Mouse lemurs were trapped in Sherman Live Traps baited with banana, marked with subcutaneously injected, individually distinctive transponders (ID-100, Trovan Small Animal Marking System, Telinject®, Römberg, Germany), and had small (1-2 mm^2^) ear biopsies taken as genetic samples. Previous generations were already marked (for methodological details regarding trapping and sampling techniques see: [42,72]). Tissue samples were stored in approximately 1 ml of Queen’s lysis buffer [73] for up to 7 months at ambient temperature in the field and then at 4°C until extraction (up to 6 years for archived samples collected in prior years) [42].

A subset of the trapped lemurs were temporarily kept in cages in the forest near the campsite to facilitate recording of vocalizations (total number trapped lemurs =107, total caged lemurs = 45). No lactating females were kept in the cages. Animals were kept either singly in cages of 0.5 m by 0.5 m by 1 m (width x depth x height) or in small groups (two to four animals) in sets of two adjoining cages, each approximately 1 m wide by 1.2 m high by 0.5 m deep. Each set of cages had two passages (0.3 m × 0.2 m × 0.2 m) connecting them. Cardboard cans were provided as nest boxes (one for each lemur) and the cages were furnished with branches for climbing. The lemurs were fed fresh fruit and could be observed catching insects that flew into the cages. They were provided with additional insects as often as possible. Water was available *ad libitum*. These housing conditions are comparable to those in captive colonies [74] and no lemurs were injured by the cages or by a cage-mate. Lemurs were released at their capture site after recording was completed (1 night – approximately 2 weeks, mean = 5 nights). Methods were approved by Madagascar National Parks (2010 permits: N102/ 10/MEF/SG/DGF/DCB.SAP/SCBSE, N103/10/MEF/SG/DGF/DCB.SAP/SCBSE, 2011 permits: N101/11/MEF/SG/DGF/DCB.SAP/SCB, N102/11/MEF/SG/DGF/DCB.SAP/SCB) and the Arizona State University Institutional Animal Care and Use Committee (Protocol: 10-1077R).

Before release, 25 adult female gray mouse lemurs (2010: n = 13, 2011: n = 15, three collared in both years, adult = 50 g) were fitted with a radio-collar (either a PicoPip or a Pip3 collar from BioTrack Ltd., United Kingdom, weight 2.3-3.1 g). We then used radio-telemetry to locate the females’ daytime sleeping sites using a TR-4 receiver (Telonics, Mesa, AZ, USA). We read the transponders of lemurs sleeping inside the nests with a handheld microchip reader (Trovan Small Animal Marking System, Telinject®, Römberg, Germany). We checked the sleeping sites on a total of 118 days (65 days in 2010, 53 days in 2011), which resulted in a range of 11-74 days of data per collared female (mean = 29 days), depending on the lifespan of the radio-collar and survival of the female.

### Recording methods and acoustic measurements

We recorded all calls given during controlled social encounters when two lemurs were introduced within the cages (or, occasionally during coincidental encounters when a free-ranging lemur outside the cage approached). The introductions inside the cages were observed and the elicited calls were considered to be agonistic when they were associated with aggressive/defensive behaviors such as fighting, chasing, fleeing, etc. When the lemurs were first introduced, the experimenter remained present during the entire night so that she could separate the lemurs if necessary. However, this was rarely necessary, and no lemurs were injured during the introductions.

We recorded the calls with a D1000X Bat Detector (flat frequency response: 5-235 kHz, sampling frequency 200 kHz, 16-bit resolution, Pettersson Elektronik, Upsala, Sweden) from a distance of approximately 2-4 meters from the inside of an observation tent. Under these conditions, agonistic calls were recorded from 15 female gray mouse lemurs. None of the lemurs were caged with female kin group members when the calls were recorded. For one additional female, calls were recorded at a distance of approximately 3 meters while she ate at a feeding platform in the forest after a conflict with another lemur.

Calls were measured in Signal 4.0 (Engineering Design) using the macro written by M. Scheumann for agonistic gray mouse lemur calls and previously used in Leliveld et al. [58]. Ten high quality calls were selected from each female. High quality calls were those that had a clearly visible fundamental frequency, low background noise, and no overlaps with other sound-producing organisms. As the calls are typically given in series, we selected 2-3 series per lemur. Each series consisted of 2-7 calls for a total of 10 calls for each of the 16 lemurs. Figure 1 and Table 4 provide a description of the acoustic parameters that were measured or calculated.

**Table 4:** Measured and calculated acoustic parameters

**Measured Parameters**	**Definition**	**Source**
*F_0_S (KHz)	Freq. of F_0_ with highest amplitude at start	Osc. & PS
*F_0_Peak (KHz)	Freq of F_0_ with highest amplitude at max of F_0_	Spect. & PS
*F_0_E (KHz)	Freq. of F_0_ with highest amplitude at end	Spect. & PS
SB_Max	Frequency at 20 dB above F_0_S	PS
SB_Min	Frequency at 20 dB below F_0_S	PS
S (ms)	Start time of call	Osc.
P (ms)	Time of highest point of F_0_	Spect.
E (ms)	End time of call	Spect.
N (ms)	Start time of next call	Osc.
**Calculated parameters**	**Definition**	**Calculation**
*Start Bandwidth (KHz)	Bandwidth of F_0_ at start	SB_Max – SB_Min
*Call Duration (ms)	Time between start and end of call	E – S
*Time to Peak (ms)	Time between start and peak of call	P – S
*Inter-call Interval (ms)	Time between end of the call and start of the next call	N – E

*Osc* Oscillogram, *PS* Power spectrum, and *Spect* Spectrogram. *parameters included in the principal component analysis. For more information see Figure 1 and Leliveld et al. [58].

### Genetic analyses

Genetic analyses were conducted at the University of Veterinary Medicine Hannover in the Institute of Zoology. Extractions were performed with a proteinase K digestion and a phenol / chloroform extraction. Eight microsatellite loci (Table 5) were successfully amplified using one of three methods: 1) We used a Qiagen Multiplex PCR Kit (Qiagen, Hilden, Germany) following the manufacturer’s instructions, but reduced to the final reaction volume to 10 μl. Ratios followed the instructions with the exception that only 1 μl of Q Solution was used. Cycling conditions followed the provided protocol with annealing temperatures of 48-58°C and up to 48 cycles. 2) We used a MyTaq DNA Polymerase kit (Bioline GmbH, Luckenwalde, Germany) following the manufacturer’s instructions and concentrations, but reducing the reaction volume to 10 μl and using 0.15 μM of each primer and 0.05 μl MyTaq. 3) We performed PCR reactions with final concentrations of 1.5-2.0 mM MgCl_2_ Solution (Invitek , Berlin, German), 1 x NH_4_-reaction buffer (50 mM Tris-HCL (pH8.8), 16 mM (NH_4_)_2_SO_4_, 0.1% Tween ©20, Invitek, Berlin, Germany) or 1 x PARR buffer (Cambio, Cambridge, UK), 225 μM of each dNTP (Fermentas Life Sciences), 0.15-0.5 μM of each primer, and 0.025 U of *Taq* DNA Polymerase. Cycling conditions for this procedure and the MyTaq kit consisted of an initial denaturation phase of 2-4 min at 92-94°C, denaturing for 20-60s at 92-94°C, annealing for 20-60s at 48-58°C, extension for 30-90s at 72°C, and a final extension phase of 5-7 min at 72°C. We used up to 48 cycles. For one marker, M3, the cycling conditions were as follows: initial denaturing at 94°C for 4 min, denaturing at 94°C for 30s, annealing at 55°C for 20s, extension at 72°C for 30s (6-7 cycles), denaturing at 94°C for 30s, annealing at 53°C for 20s, extension at 72°C for 30s (6-7 cycles), denaturing at 94°C for 30s, annealing at 50°C for 20s, extension at 72°C for 30s (25-30 cycles), and a final extension phase at 72°C for 7 min.

**Table 5:** **Characteristics of the microsatellite markers showing the number of individuals typed at each locus (N), the number of alleles observed at each locus (Alleles), expected heterozygosity (H**_**e**_**), observed heterozygosity (H**_**o**_**), the heterozygote deficit within the population (F**_**is**_**), and the P value of the heterozygote deficit**

**Marker**	**N**	**Alleles**	**H**_**e**_	**H**_**o**_	**F**_**is**_	**P**	**Citation**
M2	107	9	0.74	0.69	0.062	0.1401	[75]
M3	107	15	0.81	0.84	-0.041	0.8827	[75]
M9	106.5	16	0.90	0.89	0.010	0.4226	[75]
M10	105	24	0.93	0.90	0.041	0.0839	[75]
M21	105	11	0.83	0.70	0.155	0.0003*	[76]
M22	107	11	0.84	0.80	0.047	0.1429	[76]
M39	107	25	0.94	0.94	-0.004	0.6133	[76]
PVCA1	107	13	0.86	0.88	-0.018	0.7316	[77]
**Overall**	**106.6**	**16.1**	**0.86**	**0.85**	**0.013**	**0.1490**	-----

Significant p-values are Bonferroni corrected to be <0.00714. “Overall” shows the calculations performed across the seven loci retained in the analysis (excluding M21). The citations indicate where the primer sequences are published. The decimal N indicates individuals where only one of two alleles could be determined at that locus.

The length of the resulting PCR products were determined on an Applied Biosystems 3500 capillary sequencing machine (Applied Biosystems, Life Technologies, GmbH, Darmstadt, Germany). Alleles were scored in Genemapper 4.1 (Applied Biosystems, Life Technologies, GmbH, Darmstadt, Germany) and checked by eye. All homozygous samples were amplified at least twice, following the procedures in prior studies (i.e., [42,78]).

The mitochondrial D-loop was sequenced using the universal mammalian control region primers H16498 and L15997 [79] for all captured females. PCR was conducted in a 25 μl reaction volume with the following concentrations: 3 mM MgCl_2_, 1 × NH_4_-reaction buffer (50 mM Tris-HCL (pH8.8), 16 mM (NH_4_)_2_SO_4_, 0.1% Tween^©^20, Invitek, Berlin, Germany), 400 μM of each dNTP (Fermentas Life Sciences), 0.8 μM of each primer, 0.125 U of *Taq* DNA Polymerase. We used an initial denaturation phase of 3 min at 94°C, a denaturing phase of 1 min at 94°C, an annealing phase of 1 min at 50°C, an extension phase of 1 min at 72°C (35-50 cycles), and a final extension phase of 5 min at 72°C. For samples that did not amplify well and were weak when visualized on a 1.5% agarose gel (containing 1.3 × 10^-4^ mg/ml ethidium bromide), we used the MyTaq DNA Polymerase kit (Bioline GmbH, Luckenwalde, Germany). We followed the manufacturer’s instructions and concentrations, but reduced the reaction volume to 25 μl and used 1 μl of each primer (10 pM/μl) and 0.1 μl MyTaq. Cycling conditions were the same as above. PCR products were then cleaned using the MSB Spin PCRapace kit (Stratec Molecular GmbH, Berlin, Germany). Sequencing followed one of two procedures. We either mailed the samples to Macrogen Ltd. (http://dna.macrogen.com) where they were sequenced using an ABI 3730XL automatic DNA sequencer or we performed the sequencing reactions ourselves using the ABI Prism BigDye Terminator v. 3.1 Cycle Sequencing Kit (Applied Biosystems, Life Technologies, GmbH, Darmstadt, Germany). We used 10 μl reactions consisting of 6.5 μl cleaned PCR product, 1 μl ABI Prism BigDye Terminator Ready Reaction Mix, 2 μl 5× Sequencing Buffer, and 0.5 μl primer (10 mM) and performed 25 cycles of 96°C for 10s, 57°C for 5 s, and 60°C for 3 min. After a final cleaning step with an ethanol precipitation, subsequent sequencing was performed on an Applied Biosystems 3500 capillary sequencer.

Sequences of 446-563 bp (mean = 531.6) were edited, analyzed and aligned in SeqMan 7.0 (DNASTAR Inc., Madison, WI, USA). The final alignment and a matrix of the number of pairwise differences was calculated in Mega 5 [80], and a haplotype network was produced in Network 4.6.1.1 (Fluxus Technology Ltd., Suffolk, UK).

Sequences have been deposited in GenBank (Accession numbers: KJ183142-KJ183177).

### Relatedness calculations

Of the eight microsatellite markers, one (M21) was not in Hardy-Weinberg equilibrium and displayed a significant deficit in heterozygotes (F_is_ = 0.155, P = 0.0003, calculated in Fstat 2.9.3.2 [81]). Because this could influence the relatedness calculations, this marker was dropped from the analysis. The remaining markers and the calculations over all loci were in Hardy-Weinberg equilibrium (Table 5) and were therefore included in the relatedness calculations. While we acknowledge that increasing the number of markers improves the resolution of the kinship relationships [82], using 7 microsatellites is within the range used in similar studies on mouse lemurs (7 microsatellites in Radespiel et al. [42], 6 in Wimmer et al. [77], Radespiel et al. [78]). In addition, we maximized the genetic information obtained from these microsatellites we used by selecting markers that are highly polymorphic (9-25 alleles, see Table 2) as advocated by Harrison et al. [82].

We calculated pairwise relatedness in Kinship 1.3.1 [59] according to Queller and Goodnight [60] based on the genotypes of 107 individuals (72 males, 35 females) that were captured during the study period. We chose this relatedness estimator for two reasons. First, it has been shown to perform well on samples with a high percentage of highly related pairs [83], which we expected to have, given that we were focusing on co-sleeping females. (Prior research has shown that co-sleeping females are typically closely related [42], and indeed, in this study, all co-sleeping dyads were closely related). Second, it will allow for comparisons with previous studies on mouse lemur relatedness using this estimator (i.e., [42,84]). Using Kinship we used a simulation procedure which uses the allele frequencies within the population to test the likelihood that the r-value between each dyad was produced by a relationship of r_maternal_ = 0.5 and r_paternal_ = 0 against a null hypothesis of r_maternal_ = 0 and r_paternal_ = 0. This was performed for all possible dyads among the 107 individuals. By doing so, we distinguished between dyads with a close matrilineal relatedness and dyads that were matrilineally unrelated. This procedure is based upon Van Horn et al. [85]’s findings showing that though pairwise relatedness may not be precise enough to distinguish small differences in relatedness (e.g.., full- and half-siblings), unrelated dyads can be accurately distinguished from closely related dyads and vice versa. Van Horn et al. [85] showed that that closely related dyads are rarely misclassified as unrelated and unrelated dyads are rarely misclassified as closely related (Van Horn et al. [85], page 1177, Table 1). Kinship’s pairwise relatedness values have been shown to correlate with known pedigree relationships [85], and negatively with allelic exclusions in this population [42].

Distinguishing r_maternal_ = 0.5 and r_paternal_ = 0 from r_maternal_ = 0.0 and r_paternal_ = 0.5 was possible because we integrated the pairwise relatedness data with the mitochondrial haplotype data and co-sleeping data (discussed in greater detail below). The mitochondrial data enabled us to exclude closely related pairs with no matrilineal relationship. While we acknowledge that it could be possible for closely related paternal relatives to have the same mitochondrial haplotype, it is very unlikely that such dyads would also co-sleep as prior research on this population [42] and others [50] showed that sleeping groups consist of close matrilineal, not patrilineal, relatives.

The probability of identity between two individuals in the population was <1^-6^ , calculated according to Botstein et al. [86] in PopAssign 3.9e (written by S.M. Funk). The probabilities of exclusion, according to Jamieson and Taylor [87], calculated in PopAssign 3.9e, were 0.999941 for one parent, 1.000000 for the second parent, and 0.998505 in the case of a missing parent. Alpha was set at 0.05 for all statistical tests in this study unless otherwise specified.

Marker characteristics are shown in Table 5. Expected and observed heterozygosity (H_e_ and H_o_) for each locus and over all loci were calculated in PopAssign 3.9e. The observed F_is_ for each locus and over all loci and the associated P values testing for a deficit in heterozygotes were calculated in Fstat 2.9.3.2 [81]. The statistical error p was calculated by randomizing alleles among individuals over 7000 randomizations. P values are the proportion of randomizations that gave a larger F_is_ than the observed. The Bonferroni corrected alpha was set at <0.00714.

### Kin group selection

In order to minimize the confounding effects of paternal relatedness when testing for matrilineal signatures, we selected dyads of females within kin groups that had high pairwise relatedness and strong genetic and behavioral evidence of matrilineal relationships. While we realize that the inclusion of full sister dyads (and therefore some cases of paternal relatedness) cannot be excluded with certainty, we assume that due to the promiscuous mating system, possible multiple paternities within litters, and the high turn-over rate of mouse lemurs across field seasons [72,88,89], most of our dyads are likely to consist of mother-daughter pairs or half sisters. This would mean that on average, barring severe inbreeding, matrilineal relatedness should be much higher than patrilineal relatedness within the dyads. We grouped the dyads into matrilineal kin groups based upon three criteria. Within a kin group: 1) females had the same mitochondrial haplotype, 2) behavioral evidence showed that they co-sleep, and 3) females had a Queller and Goodnight relatedness value [60] that is significantly likely to result from a maternal relatedness of 0.5 with *all* other individuals in the group. In three out of six groups all dyads met all three criteria for kin groups. Within the remaining three groups (groups 1, 5, and 6) not all of the criteria were fulfilled for all dyads (Table 3). Within group 1, female 06-09 was not observed to share a sleeping site with the other females in her group. However, because she shared her mitochondrial haplotype with one of the other females in the group (the third could not be determined), and was closely related to both of the other two females (r = 0.61, P < 0.001 and r = 0.29, P < 0.01), 06-09 is included in the kin group. The mitochondrial haplotype of a second female (19-10) from kin group 1 was unknown, but she shared a nest and had an r-value likely to result from a maternal relatedness of 0.5 (r = 0.44, P < 0.001) with one of the other females in her group. As sleeping groups in this population have been shown to typically consist of close matrilineal relatives [42], 19-10 is also included in this matrilineal kin group. Within group 5, the two females were not observed to co-sleep, but they fulfilled the other two criteria, including having a significant r value (r = 0.36, P < 0.001) and thus are still considered a kin group. Within sleeping group 6, co-sleeping data is unavailable for two dyads. However, 17-10 co-slept with 28-09 in 2010 and with 36-11 and 46-11 in 2011. It is unknown whether 28-09 lived long enough to have the opportunity to share a sleeping site with 36-11 and 46-11 because she was not recaptured in 2011. In total, we divided the 16 females into 6 kin groups: one group of four females, two groups of three females, and three groups of two females (Table 3).

### Test of kin group signatures

In order to test whether agonistic calls are distinctive by kin group, we conducted a discriminant function analysis. We performed a principal component analysis with no rotation on the correlation matrix conducted in SPSS 21 to reduce the dimensionality of the dataset. Then, because we have a nested design (individuals are nested within kin groups), we conducted a permutated linear discriminant function analysis (pDFA) in R 2.14.0 (The R Foundation for Statistical Computing, 2011) with kin group as the test factor and individual as the control factor [90] and 10,000 permutations. As the maximum number of input parameters is one less than the number of objects in the smallest class (two individuals in some of the kin groups), we could only include one principal component in the analysis [90]. We included the first principal component because it accounted for the greatest amount of variation in the original dataset relative to the other components. Cross-validation was performed using the leave-one-out method (Mundry, R., personal communication). Because the pDFA does not produce a classification table, we present the table produced by a nonpermutated discriminant function analysis conducted in SPSS 21.

### Correlation between acoustic distance and genetic distance

We used the first principal component to calculate an acoustic distance for all dyads. First we calculated a mean value for each individual for PCA1. We then calculated the Euclidean distances between each pair of individuals producing a matrix of acoustic distances between the individuals. We conducted a Mantel test in Mantel 2.0 [91] using 1000 permutations to test for a correlation between acoustic distance and genetic relatedness.

## Competing interests

The authors declare that they have no competing interests.

## Authors’ contributions

SK designed the study, collected and analyzed the data and drafted the manuscript. UR participated in study design, supervised the genetic analyses, and assisted with data analysis and manuscript preparation. AH and LL assisted with data collection in the field. LN participated in study design, data analysis and manuscript preparation. EZ participated in study design, supervised the acoustic analyses, and assisted with data analysis and manuscript preparation. UR and EZ also provided logistical support for the fieldwork, and equipment and materials for field and lab work. All authors read and approved the final manuscript.

## Supplementary Material

Additional file 1 **The 25% quartile, median, and 75% quartile of the acoustic parameters included in the principal component analysis for each female and for each kin group (calculated across all calls for all individuals in that kin group).** Kin group values are bolded. Click here for file

Additional file 2 The percentages of each individual's calls that were correctly (green) and incorrectly (white) classified into the different kin groups using PCA1 and PCA2. Click here for file
